# Blood Levels of Indicators of Lower Respiratory Tract Damage in Chronic Bronchitis in Patients with Abdominal Obesity

**DOI:** 10.3390/diagnostics12020299

**Published:** 2022-01-25

**Authors:** Elena V. Kashtanova, Yana V. Polonskaya, Evgeniia V. Striukova, Liliia V. Shcherbakova, Evgenii A. Kurtukov, Viktoriya S. Shramko, Ekaterina M. Stakhneva, Yulia I. Ragino

**Affiliations:** Research Institute of Internal and Preventive Medicine-Branch of the Federal Research Center Institute of Cytology and Genetics, Siberian Branch of Russian Academy of Sciences (IIPM-Branch of the IC & G SB RAS), st. B.Bogatkova 175/1, 630089 Novosibirsk, Russia; elekastanova@yandex.ru (E.V.K.); stryukova.j@mail.ru (E.V.S.); 9584792@mail.ru (L.V.S.); cawertty@gmail.com (E.A.K.); nosova@211.ru (V.S.S.); stahneva@yandex.ru (E.M.S.); ragino@mail.ru (Y.I.R.)

**Keywords:** chronic bronchitis, abdominal obesity, SP-A, SP-D, α1-antitrypsin, CC16, PARC, RELM-β

## Abstract

Objective: to study biomolecules associated with pathology in the respiratory system, in particular, with the development of chronic bronchitis in patients with abdominal obesity. Materials and methods: This is a pilot study. The main group consisted of 158 people with chronic bronchitis, divided into two subgroups: one with abdominal obesity, and the other without it. The control group consisted of 68 people without chronic bronchitis. We determined the blood levels of SP-A, SP-D, α1-antitrypsin, CC16, PARC, and RELM-β. Results: In the first subgroup, patients significantly more often complained of coughing, experienced shortness of breath 1.5 times more often with light physical exertion and 2.7 times more often with moderate physical exertion. In these patients, a Tiffeneau–Pinelli index (FEV1/FVC) below 70% was 1.8 times more common, more patients had FEV1 and FVC of less than 80%, and presented a statistically significant decrease in SP-A, α1-antitrypsin, CC16 levels and an increase in PARC levels than in the second subgroup. Conclusion: In patients with chronic bronchitis and abdominal obesity, there is a decrease in the levels of SP-A, α1-antitrypsin, CC16 and an increase in the level of PARC compared with patients without abdominal obesity, which is probably due to the presence of an additional source of chronic inflammation associated with adipose tissue.

## 1. Introduction

Chronic bronchitis (CB) is a clinical phenotype in COPD and is defined as the presence of cough and sputum production for at least three months in each of two consecutive years. The pathomorphological basis of CB is epithelial metaplasia, accompanied by excessive mucus production in response to respiratory tract chronic inflammation. Studies show that in COPD, inflammation of the respiratory tract is not limited to the lungs and can go beyond it, taking the character of systemic inflammation [1,2,3,4,5,6,7]. CB can be an early marker of COPD, which in turn, affects the mortality associated with this phenotype. Recently, there have been more and more studies describing the clinical and functional features of the combined course of CB and abdominal obesity (AO), which is a predictor of an unfavorable prognosis of the disease not only for smokers but also for people who have never smoked [8,9,10]. People with a large waist circumference (WC) (110 cm or more in women and 118 cm or more in men) have been shown to have a 72% higher risk of developing COPD than people with a WC corresponding to the normal values [11]. In obese patients, structural changes in the thoracic and abdominal regions limit the mobility of the diaphragm and ribs, which affects lung ventilation. In addition, the adipose tissue is an endocrine and paracrine organ that produces numerous cytokines and bioactive mediators, causing a pro-inflammatory condition in obese patients, which is associated with an increased risk of developing a bronchopulmonary pathology [12,13]. Given the limited information in the literature on the role of biomolecules associated with pathology in the respiratory system, particularly, with the development of CB in combination with AO, research in this area is vastly relevant. This work includes the most promising biochemical markers, which, according to the available literature data, can be of diagnostic importance in the study of pulmonary pathologies, such as serum surfactant proteins A and D (SP-A, SP-D), α1-antitrypsin, protein Clara cells (CC16), pulmonary and activation-regulated chemokine (PARC), and resistin-like molecule beta (RELM-β) [14,15,16,17,18,19]. The data obtained during the study will serve as a basis for creating new laboratory panels focused on detecting bronchopulmonary disorders at early stages, which will improve their primary prevention.

The goal of this research was to study biomolecules associated with pathology in the respiratory system, particularly, with the development of chronic bronchitis in patients with abdominal obesity.

## 2. Materials and Methods

The study was conducted based on a population sample of residents of the Novosibirsk city aged 25–44 years, obtained in 2013–2016 in IIPM—Branch of the IC&G SB RAS. The design was cross-sectional. We used the database of the Territorial Compulsory Medical Insurance Fund for the Novosibirsk Region to build the sample, from which we selected 3000 people of both sexes aged 25–44 years using a random number generator. We used step-by-step epidemiological stimulation: mail invitations, phone calls, information messages in the media. During the entire period, we examined 1512 people as part of a single-stage population screening. We obtained biological material and created a database. All patients signed an informed consent to the examination and processing of personal data. This study was approved by the local Ethics Committee Protocol No. 167 of 26.11.2019.

We selected 158 people (the main group) with chronic bronchitis without exacerbation of the disease from the population sample at the time of the examination using random numbers. We formed a control group of people without chronic bronchitis (68 people), comparable in age and gender. The study included 226 people.

The non-inclusion/exclusion criteria were: exacerbation of a clinically significant severe concomitant pathology (chronic infectious and inflammatory diseases, renal and hepatic insufficiency, heart and respiratory failure), exacerbation of CB in the anamnesis at least two months before the examination, diabetes mellitus, grade 3 arterial hypertension, pregnancy.

CB was established based on anamnesis: the presence of cough and sputum production for at least three months in each of two consecutive years [20,21]. Abdominal obesity (AO) was established based on waist circumference >80 cm in women and >94 cm in men [22].

All patients with chronic bronchitis (the main group) were divided into two subgroups. The 1st subgroup included 96 patients (52.1% men and 47.9% women) with CB and AO. The 2nd subgroup included 62 patients (40.7% of men and 53.2% of women) with CB without AO. A team of doctors and nurses of IIPM—Branch of the IC & G SB RAS carried out the clinical examination of the patients. The survey program included a collection of demographic and social data, a questionnaire that identified respiratory diseases and smoking habits, a dietary questionnaire, a questionnaire on the history of chronic diseases and medication use, a 3-fold measurement of blood pressure, spirometry, anthropometry (measuring height, body weight, calculating body mass index (BMI), measuring waist and hip circumference).

Blood sampling for biochemical studies in patients was carried out from the ulnar vein in the morning, not earlier than 12 h after the last meal. We determined the levels of SP-A (CUSABIO, Wuhan, China), SP-D (BioVendor, Brno, Czech Republic), α1-antitrypsin (Immun-diagnostik AG, Bensheim, Germany), CC16 (BioVendor, Czech Republic), PARC (RayBio, Peachtree Corners, GA, USA), RELM-β (CUSABIO, Wuhan, China) in blood serum by enzyme immunoassay according to the manufacturer’s recommendations.

Statistical analyses were performed using SPSS 13.0 software. Quantitative variables for an abnormal distribution are presented as medians (Me) with an interquartile range [Q25; Q75] and for normal distribution as M ± SD. We compared groups using the Mann–Whitney criterion and the χ2 criterion. We used multivariate logistic regression for the analysis of the relationship between several independent variables; *p* < 0.05 was considered significant.

## 3. Results

At the first stage of our study, we analyzed the clinical and anamnestic data of two groups of patients, one with chronic bronchitis (the main group) and the other as a control group, and the blood levels of the studied biochemical parameters (Table 1).

The obtained data showed a significant difference in the Tiffeneau–Pinelli index (FEV1/FVC) between the studied groups. There was no difference in other parameters. Thus, the relative number of smokers and of individuals with smoking experience in the control and study groups at the time of the study did not differ, and there were no differences in the subgroups of the control group. The analysis of biochemical parameters revealed a significant difference only in the level of RELM-β. Thus, in patients with chronic bronchitis, the level of this indicator was two times higher than in the control group.

In the next stage of our study, we studied subgroups of patients with chronic bronchitis, depending on the presence or absence of abdominal obesity. The clinical and anamnestic data of the subgroups are presented in Table 2.

The clinical picture of CB was also analyzed in the study groups. It was revealed that in the group with CB and AO, in comparison with the group without AO, patients significantly more often complained of cough and experienced shortness of breath 1.5 times more often with light physical activity and 2.7 times more often with moderate physical activity (2 and 1 points on the mMRC scale, respectively). Even though the Tiffeneau–Pinelli index between the study groups did not present significant differences, 22.4% of patients with AO had a Tiffeneau–Pinelli index below 70%, while in the group without AO, the incidence of patients with a low index was 12.4%. A comparison of indicators of respiratory function showed that in the group with AO, the incidence of patients with FEV1 of at least 80% was 1.5 times higher and with FVC of less than 80% was 1.2 times higher than in the group without AO, indicating the influence of AO on lung function.

**Figure 1:**
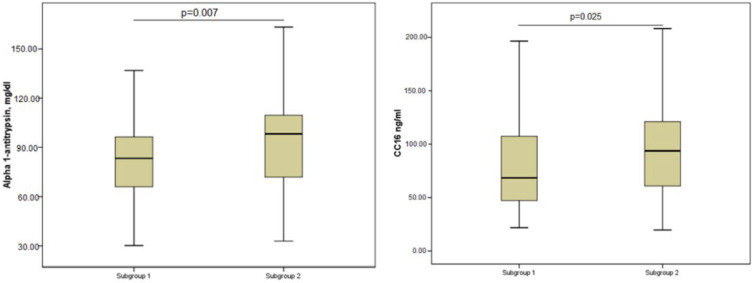

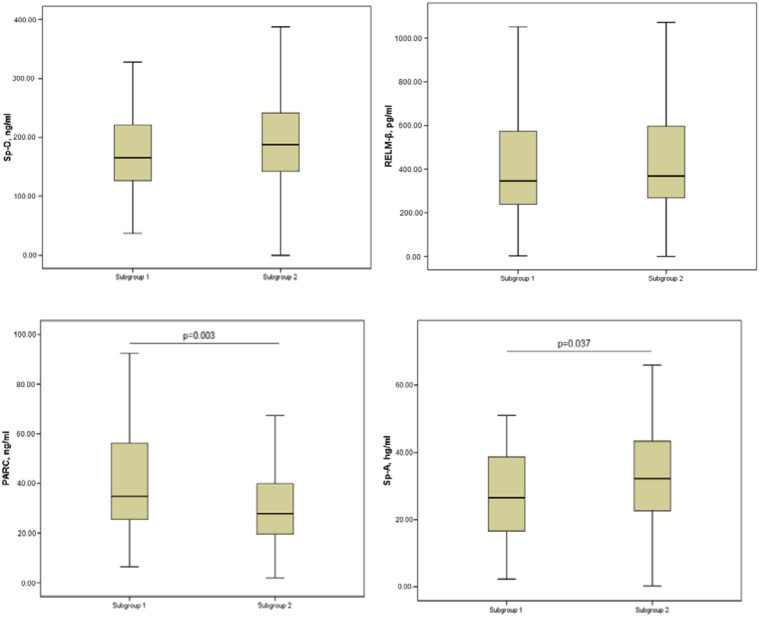
Level of the studied biochemical parameters in patients with chronic bronchitis with and without abdominal obesity.

The results of the biochemical parameters study for the group of patients with chronic bronchitis are presented in Figure 1.

We obtained statistically significant differences in the levels of the studied parameters between the first and second subgroups for SP-A, α1-antitrypsin, PARC, and CC16. The content of SP-A was 21% lower (*p* < 0.05) in patients in the first subgroup.

The level of α1-antitrypsin was also significantly lower in the first subgroup (*p* < 0.01).

PARC level was significantly higher in patients with CB and AO (*p* < 0.01).

In patients with abdominal obesity, the concentration of CC16 was statistically significantly lower (*p* < 0.05) than in the subgroup of patients without AO.

There were no statistically significant differences in the levels of RELMß and SP-D between the first and the second subgroups.

Thus, in a subgroup of patients with chronic bronchitis and abdominal obesity, there was a statistically significant decrease in the levels of such indicators as SP-A, α1-antitrypsin, and CC16 and an increase in the level of PARC.

In the next stage of statistical processing, all studied biochemical parameters were included in a logistic regression analysis model (Table 3). The presence or absence of chronic bronchitis was used as a dependent variable. All biochemical parameters were considered independent variables.

The results showed that the relative chance of developing chronic bronchitis was only associated with an increase of RELM-β level in the blood (OR = 1.01, 95% CI 1.003–1.017, *p* = 0.004).

The relative probability of having a comorbid condition was associated with an increase in blood PARC (OR = 1.029, 95% CI 1.008–1.051, *p* = 0.007) (Table 4).

## 4. Discussion

The mechanism of chronic bronchitis development is known to be based on damage to various parts of the local bronchopulmonary defense system: mucociliary clearance, local cellular and humoral immunity (the drainage function of the bronchi is disrupted; the activity of a1-antitrypsin decreases; the production of interferon, lysozyme, IgA, pulmonary surfactant decreases; the phagocytic activity of alveolar macrophages and neutrophils is inhibited). Recent observations confirm the frequent combination of respiratory diseases and visceral obesity, which is currently considered as one of several so-called “inflammatory conditions”, given that fat cells actively produce pro-inflammatory cytokines.

One of the key regulators of the functions of alveolar macrophages, the main cells of the immune system in the lungs, is SP-D, produced by non-ciliary bronchiole cells, i.e., type II alveolocytes and Clara cells [21,23,24,25]. Circulating levels of SP-D in the blood are associated with various lung diseases, including COPD, although the causal relationship remains unclear [14]. Since proteins specific to the lung epithelium, such as SP-D and SP-A, can reflect damage to the pulmonary epithelium and, as a result, increased permeability, they have been identified as potential systemic biomarkers of lung damage for various diseases, such as COPD, lung cancer, and acute respiratory distress syndrome [14,26,27]. In addition, the inverse correlation shown in studies between the concentration of serum SP-D and FEV1 indicates that serum surfactant proteins can be biomarkers of pulmonary dysfunction [28,29,30]. Jawed S. et al. [31] revealed a decrease in SP-D level in the blood in patients with COPD and obesity. SP-D is assumed to be more hydrophilic than SP-A and exits the alveolar space into the vascular compartment due to changes in the permeability of the alveolar epithelium caused by low-level chronic lung inflammation [15]. In our study, there was a statistically significant decrease in the SP-A level in patients with CB and AO, while there was no difference in the levels of SP-D between the subgroups.

Alpha-1-antitrypsin belongs to the serpin family of protease inhibitors, providing more than 90% protection against proteolytic load on the lower respiratory tract and has anti-inflammatory [32,33], immunomodulatory [34], antioxidant, bactericidal, and other properties that promote its protective effect on lung tissue [16,32,35]. Under a1-antitrypsin deficiency, an uncontrolled increase in the activity of proteolytic enzymes, primarily neutrophil elastase, occurs, as a result of which elastic fibers and other structures of the extracellular matrix of the lower respiratory tract undergo a slow destruction. This leads to a loss of elasticity in the lung tissue and to the development of obstructive disorders and emphysema. Our study showed a significant decrease in the level of α1-antitrypsin in patients with chronic bronchitis and abdominal obesity. We can only assume that a higher level of alpha-1-antitrypsin in the blood of patients without abdominal obesity is protective for lung tissues, since we did not conduct a study of the level of alpha-1-antitrypsin in the tissues of the lower respiratory tract. Smoking plays a significant role in lung damage in the presence of a deficiency of α1-antitrypsin. Cigarette smoke can further enhance the polymerization of α1-antitrypsin molecules, disrupt elastin synthesis in the lungs, and support neutrophilic inflammation [36,37]. According to our results, the level of α1-antitrypsin was lower in smoking patients (*p* = 0.013). There were no associations of other studied indicators with smoking.

PARC is a CC chemokine and is highly expressed in the lungs and in antigen-presenting cells, such as macrophages and dendritic cells, which can act as chemoattractants for both lymphocytes and immature dendritic cells [38]. In tissues, it is mainly associated with conditions including fibrosis and inflammation. Thus, CCL18 induces pulmonary fibrosis by stimulating alveolar fibroblasts to produce collagen [39], and CCL18 levels in blood plasma are a marker of disease activity [17,40]. Studies show that serum PARC levels are elevated in patients with COPD and are associated with clinical and functional outcomes and mortality associated with this disease [41,42]. Dilektasli A. et al. [43] demonstrated a significant association of the level of PARC in blood serum with the frequency of exacerbations, especially with severe exacerbations of COPD requiring hospitalization. Our study showed an increase of PARC levels in patients with chronic bronchitis and abdominal obesity and also revealed the association of PARC with abdominal obesity. Similar data were obtained in studies by Hägg D.A. et al., which showed an association of CCL18 levels in blood serum with body weight, waist circumference, and waist-to-hip ratio, but not with body mass index [44]. Hogling et al. [45] showed a relationship between CCL18 levels in adipose tissue and blood and metabolic risk factors in women. The authors showed that this protein, secreted by adipocytes, is significantly more strongly associated with metabolic risk factors than pro-inflammatory cytokines such as TNF-or IL-6 and may play a causal role in determining metabolic phenotypes in humans.

Recent studies indicate that Clara cells play a role in body protection, have an immunomodulatory effect, and participate in the remodeling of the respiratory tract through the production of specific factors, such as CC16 [46]. Several researchers have shown that in asthma and COPD patients, there is a significant deficiency of CC16 in blood and in the respiratory tract [18,47]. Studies of the Lovelace Smokers cohort have shown that plasma CC16 levels are associated with CKD and decreased lung function even before patients develop symptoms of CKD [48]. A decrease in the expression of CC16 in the lungs or epithelial cells producing CC16 may be an etiological factor of lung diseases or a predictor of it [18]. In our study, a decrease in CC16 was recorded in the group of patients with AO, especially in women, which may be associated with a greater intensity of inflammatory changes in the bronchopulmonary system linked to the presence of obesity. The obtained results are consistent with the literature data, according to which AO is associated with the appearance of respiratory symptoms and is another source of systemic inflammation in CB and endocrine and metabolic disorders, which worsen the clinical course and prognosis of patients with this pathology [12,13,49].

Resistin-like molecule-β (RELM-β) is a protein belonging to the RELM/FIZZ family of secretory proteins rich in cysteine, having homology with resistin. RELM-β was detected in structural cells (epithelial, fibroblast, and smooth muscle cells of the bronchi) of the human respiratory tract in the context of scleroderma-associated pulmonary hypertension, which is consistent with the possible role of this protein in vascular remodeling [19]. Studies by Fang C. L. et al. have shown that RELM-β is produced in excess in patients with asthma and can play a significant functional role in the remodeling of the respiratory tract [50,51]. Our study did not show a difference in the level of this indicator in subgroups of patients with chronic bronchitis, depending on the presence of abdominal obesity. However, a statistically significant difference was found when comparing the control group and patients with chronic bronchitis. The obtained results analysis showed that with an increase in the blood level of RELM-β, the chance of developing chronic bronchitis increased.

This study has several limitations. It is a pilot study, so the sample was small. Based on the obtained results, we plan to continue research in this area with an enlargement of the sample. We did not carry out genotyping of the SERPINA1 gene encoding the alpha-1-antitrypsin protein and, accordingly, we could not identify patients with a genetically determined low level of this indicator.

## 5. Conclusions

In patients with chronic bronchitis and abdominal obesity, there is a decrease in the levels of biomolecules that are protective in the lower respiratory tract and an increase in the level of PARC, compared with patients without abdominal obesity, which is probably due to the presence of an additional source of chronic inflammation associated with adipose tissue. The pathogenesis of this combined condition remains unexplored and requires further research.

## Figures and Tables

**Table 1:** Clinical, anamnestic, and biochemical data of patients in the main and control groups (M ± SD).

Parameters	Main Group n = 158	Control Group n = 68	*p*
Age, years	35.44 ± 6.38	35.42 ± 5.89	0.931
Waist circumference, cm	84.38 ± 12.7	86.47 ± 13.78	0.264
SBP, mmHg	118.26 ± 14.36	120.21 ± 15.76	0.332
DBP, mmHg	76.33 ± 10.02	78.92 ± 11.22	0.078
BMI, kg/m^2^	25.45 ± 5.06	26.25 ± 6.51	0.588
BMI ≥ 30, %	20.4	22.1	0.322
Smoking, %	35. 9	45.3	0.894
Heart Rate	73.86 ± 11.06	74.54 ± 13.15	0.595
FEV1/FVC	76.83 ± 9.73	80.04 ± 6.17	0.013
Sp-D, ng/mL	171.7 (130.22; 230.73)	140.2 (73.55; 179.22)	0.203
Sp-A, ng/mL	29.86 (19.81; 40.59)	33.47 (28.86; 38.72)	0.492
A1-Antitrypsin, mg/dL	86.81 (67.14; 103.84)	73.48 (63.08; 92.03)	0.062
PARC, ng/mL	31.56 (23.61; 61.44)	35.33 (22.34; 44.58)	0.882
CC16, ng/mL	76.28 (53.96; 116.52)	93.47 (69.32; 102.6)	0.438
RELMß, pg/mL	350.5 (241.58; 593.61)	167.54 (102.28; 263.03)	0.0001

**Table 2:** Clinical and anamnestic data of patients in the first and second subgroups (M ± SD).

Parameters.	Subgroup 1			Subgroup 2		
	Men n = 50	Women n = 46	Total n = 96	Men n = 33	Women n = 29	Total n = 62
Age, years	37.67 ± 5.87	38.62 ± 5.63	38.12 ± 5.74	36.51 ± 5.37	36.58 ± 5.82	36.54 ± 5.54
Waist circumference, cm	106.1 ± 12.05 *	91.44 ± 10.04 *	99.07 ± 13.3 *	84.24 ± 6.44	72.72 ± 4.92	78.86 ± 8.15
SBP, mmHg	130.76 ± 12.11	122.29 ± 18.65 *	126.7 ± 14.92 *	126.18 ± 11.34	111.07 ± 9.32	119.11 ± 12.85
DBP, mmHg	86.01 ± 10.11	79.52 ± 11.09 *	82.9 ± 11.03 *	83.38 ± 10.09	73.79 ± 7.42	78.89 ± 10.1
Heart Rate	76.29 ± 10.31	72.87 ± 10.79	74.65 ± 10.63	72.91 ± 12.08	75.14 ± 10.18	73.95 ± 11.12
BMI, kg/m^2^	32.0 ± 4.77 *	29.3 ± 4.63 *	30.69 ± 4.87 *	23.4 ± 3.0	22.03 ± 2,42	22.76 ± 2.8
BMI ≥ 30, %	60.0	34.8	47.9	0	0	0
Smoking, abs., % in subgroup	58.1	25.9	40.2	42.9	20.9	26.3
FEV1/FVC	76.0 ± 11.96	76.71 ± 9.19	76.34 ± 10.67	77.74 ± 6.73	77.44 ± 9.54	77.6 ± 8.1
FEV1 % pred	93.71 ± 14.2	86.38 ± 16.41	89.68 ± 15.81	92.56 ± 16.33	92.44 ± 13.53	92.46 ± 14.01
FVC % pred	97.57 ± 13.59	94.41 ± 13.13	95.83 ± 13.37	93.87 ± 11.95	98.08 ± 16.27	97.06 ± 15.35

Footnote: *—*p* < 0.01 in comparison with the second subgroup.

**Table 3:** Logistic regression analysis of the relative chance of developing bronchitis.

Parameters	Exp(B)	95.0% C.I. for Exp(B)		*p*
		Lower	Upper	
Sp-D	0.997	0.987	1.008	0.580
Sp-A	0.971	0.916	1.029	0.322
α1-antitrypsin	1.043	0.975	1.116	0.217
PARC	0.972	0.921	1.026	0.303
CC16	0.998	0.970	1.025	0.860
RELM-β	1.020	1.007	1.034	0.003
sex	0.14	0.012	1.709	0.124
age	1.251	0.982	1.592	0.104
smoke	1.675	0.704	3.984	0.244

**Table 4:** Logistic regression analysis of the relative chance of developing a comorbid state in patients with chronic bronchitis.

Parameters	Exp(B)	95.0% C.I. for Exp(B)		*p*
		Lower	Upper	
Sp-D	0.999	0.995	1.003	0.561
Sp-A	0.997	0.974	1.020	0.770
α1-antitrypsin	0.995	0.990	1.001	0.116
PARC	1.029	1.008	1.051	0.007
CC16	0.998	0.993	1.002	0.334
RELM-β	0.999	0.997	1.000	0.132
sex	1.679	0.759	3.716	0.201
age	0.991	0.932	1.054	0.777
smoke	1.675	0.704	3.984	0.244

## References

[1] Gan W.Q., Man S.F., Senthilselvan A., Sin D.D. (2004). Association between chronic obstructive pulmonary disease and systemic inflammation: A systematic review and a meta-analysis. Thorax.

[2] Wouters E.F. (2005). Local and Systemic Inflammation in Chronic Obstructive Pulmonary Disease. Proc. Am. Thorac. Soc..

[3] Walter R.E., Wilk J.B., Larson M.G., Vasan R.S., Keaney J.F., Lipinska I., O’Connor G., Benjamin E. (2008). Systemic Inflammation and COPD: The Framingham Heart Study. Chest.

[4] Agusti A. (2007). Systemic Effects of Chronic Obstructive Pulmonary Disease: What We Know and What We Don’t Know (but Should). Proc. Am. Thorac. Soc..

[5] Sevenoaks M.J., Stockley R.A. (2006). Chronic Obstructive Pulmonary Disease, inflammation and co-morbidity—A common inflammatory phenotype?. Respir. Res..

[6] Fabbri L.M., Rabe K.F. (2007). From COPD to chronic systemic inflammatory syndrome?. Lancet.

[7] Sin D.D., Man S.F. (2007). Systemic inflammation and mortality in chronic obstructive pulmonary disease. Can. J. Physiol. Pharmacol..

[8] Makker H., Zammit C., Liddicoat H., Moonsie I. (2010). Obesity and respiratory diseases. Int. J. Gen. Med..

[9] Boikov V.A., Kobyakova O.S., Deev I.A., Kulikov E.S., Starovoitova E.A. (2013). The state of external respiratory function in obese patients. Bûlletenʹ Sibirskoj Mediciny.

[10] Foumani A.A., Neyaragh M.M., Ranjbar Z.A., Leyli E.K., Ildari S., Jafari A. (2019). Waist Circumference and Spirometric Measurements in Chronic Obstructive Pulmonary Disease. Osong Public Health Res. Perspect..

[11] Behrens G., Matthews C.E., Moore S.C., Hollenbeck A.R., Leitzmann M.F. (2014). Body size and physical activity in relation to incidence of chronic obstructive pulmonary disease. Can. Med Assoc. J..

[12] Akpinar E.E., Akpınar S., Ertek S., Sayin E., Gulhan M. (2012). Systemic inflammation and metabolic syndrome in stable COPD patients. Tuberkuloz ve Toraks.

[13] Vujic T., Nagorni O., Maric G., Popovic L., Jankovic J. (2016). Metabolic syndrome in patients with chronic obstructive pulmonary disease: Frequency and relationship with systemic inflammation. Hippokratia.

[14] Ju C.-R., Liu W., Chen R.-C. (2012). Serum surfactant protein D: Biomarker of chronic obstructive pulmonary disease. Dis. Markers.

[15] Eisner M.D., Parsons P., Matthay M.A., Ware L., Greene K. (2003). Acute Respiratory Distress Syndrome Network. Plasma surfactant protein levels and clinical outcomes in patients with acute lung injury. Thorax.

[16] Pervakova M.Y., Titova O.N., Shumilov A.A., Lapin S.V., Surkova E.A., Emanuel V.L. (2016). Features of respiratory function in-dicators in patients with chronic obstructive pulmonary disease with alpha-1-antitrypsin deficiency. Med. Counc..

[17] Prasse A., Probst C., Bargagli E., Zissel G., Toews G.B., Flaherty K.R., Olschewski M., Rottoli P., Müller-Quernheim J. (2009). Serum CC-Chemokine Ligand 18 Concentration Predicts Outcome in Idiopathic Pulmonary Fibrosis. Am. J. Respir. Crit. Care Med..

[18] Guerra S., Vasquez M.M., Spangenberg A., Halonen M., Martin R.J. (2016). Club cell secretory protein in serum and bronchoalveolar lavage of patients with asthma. J. Allergy Clin. Immunol..

[19] Angelini D.J., Su Q., Yamaji-Kegan K., Fan C., Teng X., Hassoun P.M., Yang S.C., Champion H.C., Tuder R.M., Johns R.A. (2009). Resistin-Like Molecule-beta in Scleroderma-Associated Pulmonary Hypertension. Am. J. Respir. Cell Mol. Biol..

[20] Stuart-Harris C.H., Crofton J., Gilson J.C., Gough J., Holland W., Knowelden J., Lawther P.J., Mckerrow C.B., Morris J.N., Oswald N.C. (1965). Definition and classification of chronic bronchitis for clinical and epidemiological purposes. A report to the Medical Research Council by their Committee on the Aetiology of Chronic Bronchitis. Lancet.

[21] Kishore U., Greenhough T.J., Waters P., Shrive A.K., Ghai R., Kamran M.F., Bernal A.L., Reid K.B., Madan T., Chakraborty T. (2006). Surfactant proteins SP-A and SP-D: Structure, function and receptors. Mol. Immunol..

[22] Yumuk V., Tsigos C., Fried M., Schindler K., Busetto L., Micic D., Toplak H. (2015). Obesity Management Task Force of the European Association for the Study of Obesity. Obes. Facts.

[23] Orgeig S., Hiemstra P.S., Veldhuizen E.J., Casals C., Clark H.W., Haczku A., Knudsen L., Possmayer F. (2010). Recent advances in alveolar biology: Evolution and function of alveolar proteins. Respir. Physiol. Neurobiol..

[24] Lyamina S.V., Vedenikin T.Y., Malyshev I.Y. (2011). A modern approach to the analysis of the immune response in lung diseases: Surfactant protein D and its role. Sovremennyye Problemy Nauki i Obrazovaniya.

[25] Hartl D., Griese M. (2006). Surfactant protein D in human lung diseases. Eur. J. Clin. Investig..

[26] Lomas D.A., Silverman E.K., Edwards L.D., Locantore N.W., Miller B.E., Horstman D.H., Tal-Singer R. (2009). Evaluation of COPD Longitudinally to Identify Predictive Surrogate Endpoints study investigators Serum surfactant protein D is steroid sensitive and associated with exacerbations of COPD. Eur. Respir. J..

[27] Shiels M.S., Chaturvedi A.K., Katki H.A., Gochuico B.R., Caporaso N.E., Engels E.A. (2011). Circulating Markers of Interstitial Lung Disease and Subsequent Risk of Lung Cancer. Cancer Epidemiol. Biomarkers Prev..

[28] Sin D.D., Leung R., Gan W.Q., Man S.P. (2007). Circulating surfactant protein D as a potential lung-specific biomarker of health outcomes in COPD: A pilot study. BMC Pulm. Med..

[29] Winkler C., Atochina-Vasserman E.N., Holz O., Beers M.F., Erpenbeck V.J., Krug N., Roepcke S., Lauer G., Elmlinger M., Hohlfeld J.M. (2011). Comprehensive characterisation of pulmonary and serum surfactant protein D in COPD. Respir. Res..

[30] Shakoori T.A., Sin D.D., Ghafoor F., Bashir S., Bokhari S.N. (2009). Serum surfactant protein D during acute exacerbations of chronic obstructive pulmonary disease. Dis. Markers.

[31] Jawed S., Mannan N., Qureshi M.A. (2017). Association of Surfactant Protein-D With Obesity. J. Ayub Med Coll. Abbottabad.

[32] Bergin D.A., Hurley K., McElvaney N.G., Reeves E.P. (2012). Alpha-1 Antitrypsin: A Potent Anti-Inflammatory and Potential Novel Therapeutic Agent. Arch. Immunol. Ther. Exp. (Warsz).

[33] Bergin D.A., Reeves E.P., Meleady P., Henry M., McElvaney O.J., Carroll T.P., Condron C., Chotirmall S.H., Clynes M., O’Neill S.J. (2010). α-1 Antitrypsin regulates human neutrophil chemotaxis induced by soluble immune complexes and IL-8. J. Clin. Investig..

[34] Serban K.A., Petrache I. (2016). Alpha-1 Antitrypsin and Lung Cell Apoptosis. Ann. Am. Thorac. Soc..

[35] Gembitskaya T.E., Chermensky A.G., Ilkovich M.M., Tsamprubi S. (2014). Primary pulmonary emphysema in a young man caused by homozygous deficiency of α1- antitrypsin (genotype ZZ): Prospects for organizing patient care. Pulmonology.

[36] Osman M., Cantor J.O., Roffman S., Keller S., Turino G.M., Mandl I. (1985). Cigarette smoke impairs elastin resynthesis in lungs of hamsters with elastase-induced emphysema. Am. Rev. Respir. Dis..

[37] Morrison H.M., Kramps J.A., Burnett D., Stockley R.A. (1987). Lung lavage fluid from patients with α-1 proteinase inhibitor deficiency or chronic obstructive bronchitis: Anti-elastase function and cell profile. Clin. Sci..

[38] Tsicopoulos A., Chang Y., Yahia S.A., de Nadai P., Chenivesse C. (2012). Role of CCL18 in asthma and lung immunity. Clin. Exp. Allergy.

[39] Prasse A., Pechkovsky D.V., Toews G.B., Jungraithmayr W., Kollert F., Goldmann T., Vollmer E., Müller-Quernheim J., Zissel G. (2006). A Vicious Circle of Alveolar Macrophages and Fibroblasts Perpetuates Pulmonary Fibrosis via CCL18. Am. J. Respir. Crit. Care Med..

[40] Tiev K.P., Hua-Huy T., Kettaneh A., Gain M., Duong-Quy S., Tolédano C., Cabané J., Dinh-Xuan A.T. (2011). Serum CC chemokine ligand-18 predicts lung disease worsening in systemic sclerosis. Eur. Respir. J..

[41] Sin D.D., Miller B.E., Duvoix A., Man S.F.P., Zhang X., Silverman E.K., Connett J.E., Anthonisen N.A., Wise R.A., Tashkin D. (2011). Serum PARC/CCL-18 Concentrations and Health Outcomes in Chronic Obstructive Pulmonary Disease. Am. J. Respir. Crit. Care Med..

[42] Pinto-Plata V., Casanova C., Müllerova H., de Torres J.P., Corado H., Varo N., Cordoba E., Zeineldine S., Paz H., Baz R. (2012). Inflammatory and repair serum biomarker pattern. Association to clinical outcomes in COPD. Respir. Res..

[43] Dilektasli A.G., Demirdogen Cetinoglu E., Uzaslan E., Budak F., Coskun F., Ursavas A., Ercan I., Ege E. (2017). Serum CCL-18 level is a risk factor for COPD exacerbations requiring hospitalization. Int. J. Chronic Obstr. Pulm. Dis..

[44] Hägg D.A., Olson F.J., Kjelldahl J., Jernås M., Thelle D.S., Carlsson L.M., Fagerberg B., Svensson P.-A. (2009). Expression of chemokine (C–C motif) ligand 18 in human macrophages and atherosclerotic plaques. Atherosclerosis.

[45] Eriksson Hogling D., Petrus P., Gao H., Bäckdahl J., Dahlman I., Laurencikiene J., Acosta J., Ehrlund A., Näslund E., Kulyté A. (2016). Adipose and Circulating CCL18 Levels Associate with Metabolic Risk Factors in Women. J. Clin. Endocrinol. Metab..

[46] Broeckaert F., Clippe A., Knoops B., Hermans C., Bernard A. (2000). Clara Cell Secretory Protein (CC16): Features as a Peripheral Lung Biomarker. Ann. N. Y. Acad. Sci..

[47] Laucho-Contreras M.E., Polverino F., Tesfaigzi Y., Pilon A., Celli B.R., Owen C.A. (2016). Club Cell Protein 16 (CC16) Augmentation: A Potential Disease-modifying Approach for Chronic Obstructive Pulmonary Disease (COPD). Expert Opin. Ther. Targets.

[48] Petersen H., Leng S., Belinsky S.A., Miller B.E., Tal-Singer R., Owen C.A., Celli B., Tesfaigzi Y. (2015). Low plasma CC16 levels in smokers are associated with a higher risk for chronic bronchitis. Eur. Respir. J..

[49] Chuchalin A.G., Tseymakh I.Y., Momot A.P., Mamaev A.N., Karbyshev I.A., Kostyuchenko G.I. (2014). Changes in systemic inflammatory and hemostatic response in patients with co-morbidity of exacerbation of chronic obstructive pulmonary disease, chronic heart failure and obesity. Russ. Pulmonol..

[50] Fang C.L., Yin L.J., Sharma S., Kierstein S., Wu H.F., Eid G., Haczku A., Corrigan C.J., Ying S. (2014). Resistin-like molecule-β (RELM-β) targets airways fibroblasts to effect remodelling in asthma: From mouse to man. Clin. Exp. Allergy.

[51] LeMessurier K., Palipane M., Tiwary M., Gavin B., Samarasinghe A.E. (2018). Chronic features of allergic asthma are enhanced in the absence of resistin-like molecule-beta. Sci. Rep..

